# Downregulation of annexin A3 inhibits tumor metastasis and decreases drug resistance in breast cancer

**DOI:** 10.1038/s41419-017-0143-z

**Published:** 2018-01-26

**Authors:** Ruikai Du, Bingjie Liu, Lei Zhou, Dong Wang, Xueyan He, Xiaojun Xu, Lixing Zhang, Chaoshi Niu, Suling Liu

**Affiliations:** 1 The CAS Key Laboratory of Innate Immunity and Chronic Disease, Hefei National Laboratory for Physical Sciences at the Microscale, School of Life Science and Medical Center, University of Science & Technology of China, Hefei, Anhui 230027 China; 2 Key Laboratory of Breast Cancer in Shanghai, Cancer Institute, Department of Breast Surgery; Institutes of Biomedical Sciences; Innovation Center for Cell Signaling Network; Fudan University Shanghai Cancer Center, Shanghai, 200032 China; 3 The Department of Breast Surgery, The First Affiliated Hospital, Anhui Medical University, Hefei, Anhui 230032 China; 4 Department of Neurosurgery, Anhui Provincial Hospital Affiliated to Anhui Medical University, Hefei, Anhui 230001 China; 5 AnHui Province Key Laboratory of Brain Function and Brain Disease, Hefei, Anhui 230001 China

## Abstract

Annexin A3 (*ANXA3*) is dysregulated and plays an important role in various cancers. However, the role of *ANXA3* in breast cancer is still unclear. Here, we observed that the expression level of *ANXA3* was significantly upregulated in breast cancer tissues. ANXA3 knockdown inhibited cell invasion but promoted cell proliferation in both in vitro and in vivo assays. Furthermore, we found that ANXA3 knockdown inhibited the NFκB pathway via upregulating IκBα, resulting in mesenchymal–epithelial transition (MET) and a heterogeneity change of breast cancer stem cells (BCSCs). In addition, we demonstrated that ANXA3 knockdown increased the sensitivity of breast cancer cells to doxorubicin by increasing the drug uptake. The combination of ANXA3 knockdown and doxorubicin treatment simultaneously inhibited tumor growth and metastasis in vivo. This study described the role and mechanisms of ANXA3 in regulating BCSCs and breast cancer growth and metastasis, indicating that downregulating ANXA3 together with chemotherapy might be a novel therapeutic strategy for treating breast cancer.

## Introduction

Breast cancer is the most common malignancy in women and is a serious threat to women’s health^1^. Similar to other cancers, in breast cancer, metastasis accounts for the vast majority of breast cancer deaths^2^. Despite advances in cancer diagnosis and treatment in recent years, traditional treatments (radiotherapy, chemotherapy, and hormone therapy) are always limited by the resistance of some tumor cells^3^, thus forcing researchers to continue to look for new therapeutic approaches and targets.

Epithelial–mesenchymal transition (EMT) is a process of epithelial cells losing their cell polarity and cell adhesion and acquiring invasive properties to become mesenchymal cells. E-cadherin and Vimentin are considered markers of epithelial cells and mesenchymal cells, respectively^4^. EMT is necessary for the development of embryos^5^ and has been proven to play an important role in tumor metastasis and drug resistance^6,7^. Several important signaling pathways (Wnt/β-catenin, MAPK and NF-κB) are involved in EMT and correlated with tumor progression^8–10^.

In recent years, the cancer stem cell (CSC) hypothesis has been proposed, suggesting that CSCs play a decisive role in the development and progression of multiple cancers^11–14^ and are responsible for the recurrence of cancer due to their strong tolerance to traditional chemotherapies^15^. Therefore, clarifying the regulation mechanisms of CSCs is critical for developing more effective therapies for cancers. Our previous research showed breast cancer stem cells (BCSCs) have heterogeneity and exist in a distinct, invasive, mesenchymal-like state marked by CD24^−^/CD44^+^ and a proliferative, epithelial-like state marked by high aldehyde dehydrogenase activity (ALDH^+^)^16^. Despite our growing understanding of the importance of and complexity of BCSCs, the mechanisms of BCSC regulation remain limited.

Annexin A3 (ANXA3) is a member of the annexin family, which can bind to acidic phospholipids in a calcium-dependent manner^17^. ANXA3 has a role in cell differentiation, cell migration, immune regulation, and bone formation^18^. In recent years, it has been shown that ANXA3 plays a role in a variety of tumor development processes. Overexpression of ANXA3 promotes tumor proliferation and metastasis in lung, liver, and ovarian carcinoma^19–22^ and is associated with chemotherapy resistance^22–24^. The latest research shows that ANXA3 is highly expressed in CD133^+^ liver CSCs and plays a regulatory function^25,26^. However, the function of ANXA3 in breast cancer remains to be elucidated, and the effect of ANXA3 on regulating BCSCs has not been investigated.

In this study, we found that ANXA3 is significantly upregulated in breast tumor tissues from clinical biopsies. ANXA3 knockdown suppressed breast cancer cell invasion but promoted proliferation both in vitro and in vivo, which was due to the IκBα-mediated mesenchymal–epithelial transition and the switch of different states of BCSCs. In addition, we also showed that ANXA3 knockdown promoted the uptake of doxorubicin, and the inhibition of ANXA3 in combination with doxorubicin could efficiently lead to blocking tumor growth as well as tumor metastasis.

## Results

### *ANXA3* is upregulated in breast cancer tissues and is positively correlated with poor overall survival

**Fig. 1:**
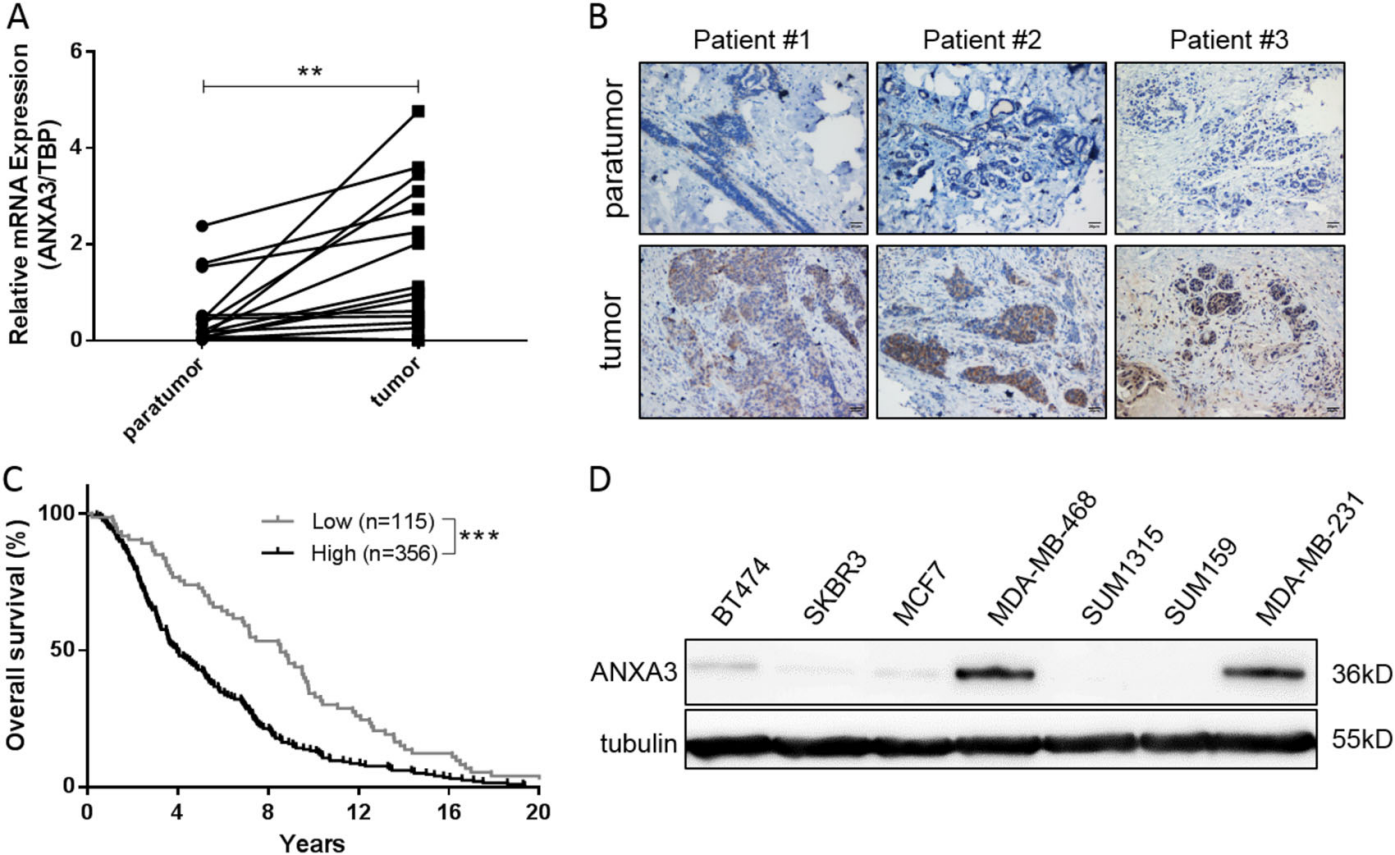
ANXA3 is upregulated in breast cancer tissues and correlated with poor overall survival **a** The mRNA expression level of ANXA3 was examined in clinical breast tumor tissues and paratumor tissues by qRT-PCR. The two connected dots represent the ANXA3 levels in the tumor and paratumor from the same patient (*n* = 16). **p < 0.01. **b** The ANXA3 protein level in clinical breast tumor tissues and paratumor tissues are shown by immunohistochemistry (Brown: ANXA3). **c** Kaplan–Meier survival curves of breast cancer patients (log-rank test, ***p < 0.001), data from the Oncomine database (*n* = 471). **d** The ANXA3 protein level was measured in several breast cancer cell lines by western blotting

To determine the expression pattern of ANXA3 in breast cancer samples, 16 pairs of breast cancer tissues and their corresponding adjacent normal tissues were analyzed for *ANXA3* expression using quantitative reverse transcription quantitative real-time PCR (qRT-PCR). The results showed that *ANXA3* was significantly upregulated in tumor tissues (Fig. 1a). At the same time, the protein level of ANXA3 was verified by immunohistochemistry (IHC; Fig. 1b). To further investigate the correlation between *ANXA3* expression and the patients’ overall survival, we analyzed data from 471 patients in the Oncomine database. Kaplan–Meier analysis indicated that patients with low ANXA3 expression had a longer overall survival (Fig. 1c). These results suggest that upregulation of ANXA3 is associated with poor prognosis in breast cancer, indicating the ANXA3 might be a good predictor of prognosis for breast cancer patients.

### *ANXA3* knockdown inhibits cell invasion but promotes cell proliferation in vitro via inducing mesenchymal–epithelial transition

**Fig. 2:**
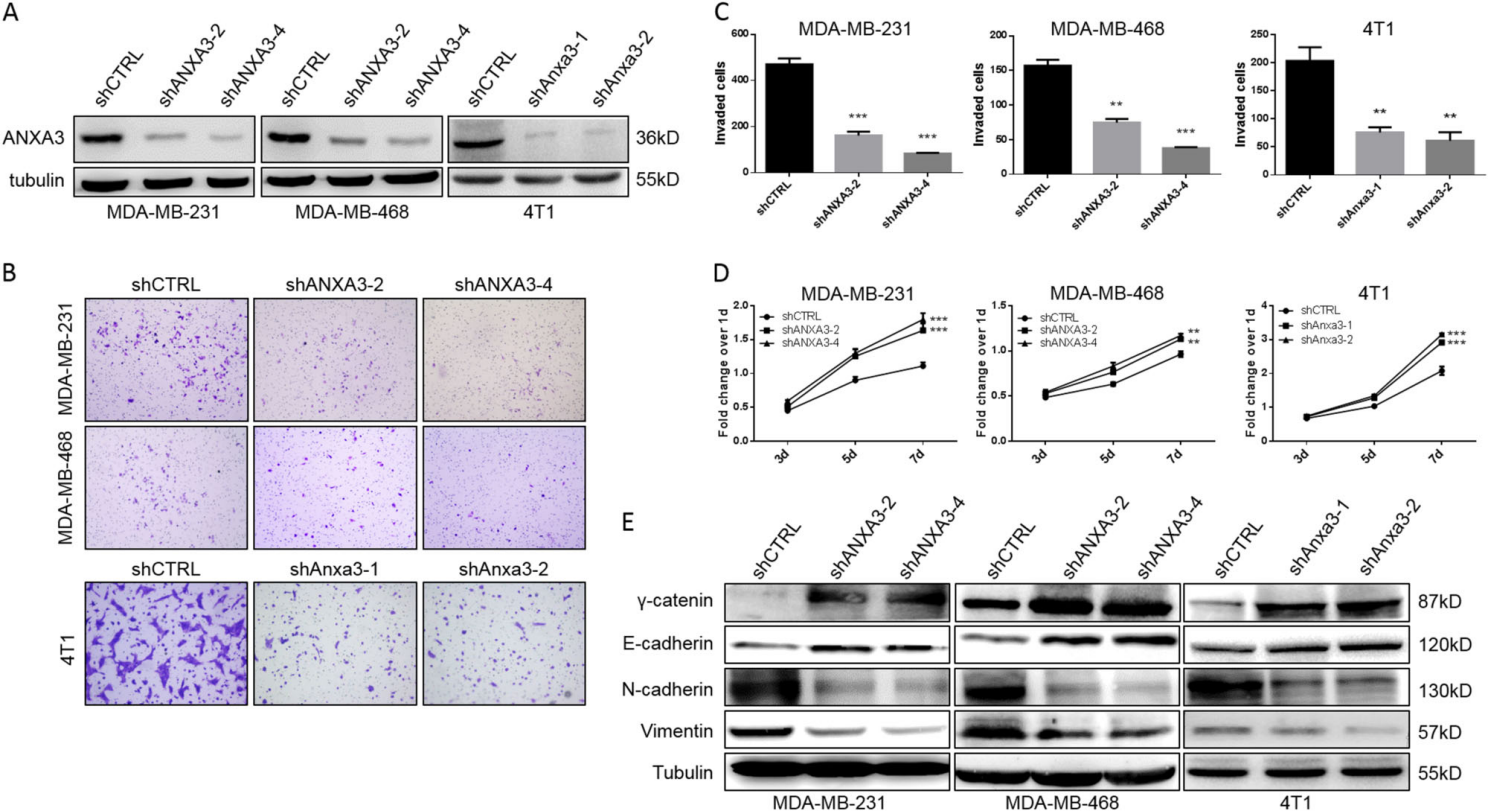
ANXA3 knockdown significantly inhibits in vitro cell invasion but promotes cell proliferation, as well as induces an epithelial phenotype **a** Human ANXA3 or mouse Anxa3 was knocked down in human breast cancer cell lines MDA-MB-231 and MDA-MB-468 or mouse mammary tumor cell line 4T1 via infection with shCTRL, shANXA3, or shAnxa3 lentivirus. ANXA3 or Anxa3 expression was detected by western blotting. **b** Cell invasion ability was measured by the Matrigel invasion as described in the methods. **c** Quantitative analysis of the total invaded cells in **b**. **p < 0.01; ***p < 0.001. **d** Cell proliferation activity was measured using an MTT assay as described in the methods. **p < 0.01; ***p < 0.001. **e** The protein expression levels of E-cadherin, γ-catenin, Vimentin, and N-cadherin were detected by western blotting

To examine the function of *ANXA3* in breast cancer, we analyzed *ANXA3* expression in different human breast cancer cell lines by western blot. We showed that MDA-MB-231 and MDA-MB-468 had markedly higher ANXA3 protein levels (Fig. 1d). To explore the effect of *ANXA3* on breast cancer cells, we established *ANXA3-*knockdown cell lines using MDA-MB-231, MDA-MB-468, and mouse mammary cancer cell line 4T1. Each cell line was infected by lentivirus with two specific short hairpin RNA (shRNA) sequence which targeted *ANXA3* mRNA and a random sequence shRNA as the control. Both of the specific shRNAs significantly decrease the *ANXA3* expression level (Fig. 2a). In the Matrigel transwell assay, *ANXA3* knockdown inhibited cell invasion significantly (Fig. 2b, c). Interestingly, utilizing the MTT cell proliferation assay, we showed that *ANXA3* knockdown accelerated cell proliferation significantly (Fig. 2d). Our previous study demonstrates that in breast tumors, mesenchymal-like BCSCs are relatively quiescent and have a highly invasive capacity, whereas the epithelial-like BCSCs are more proliferative^16^. To determine whether *ANXA3* knockdown induced mesenchymal–epithelial transition (MET), we analyzed the expression of epithelial markers E-cadherin and γ-catenin and mesenchymal markers Vimentin and N-cadherin by western blotting. Those results showed that *ANXA3* knockdown decreased mesenchymal marker expression but increased epithelial marker expression (Fig. 2e), which suggested that *ANXA3* knockdown induced the MET process in breast cancer cell lines.

### *ANXA3* knockdown inhibits metastasis but promotes tumor growth in vivo

**Fig. 3:**
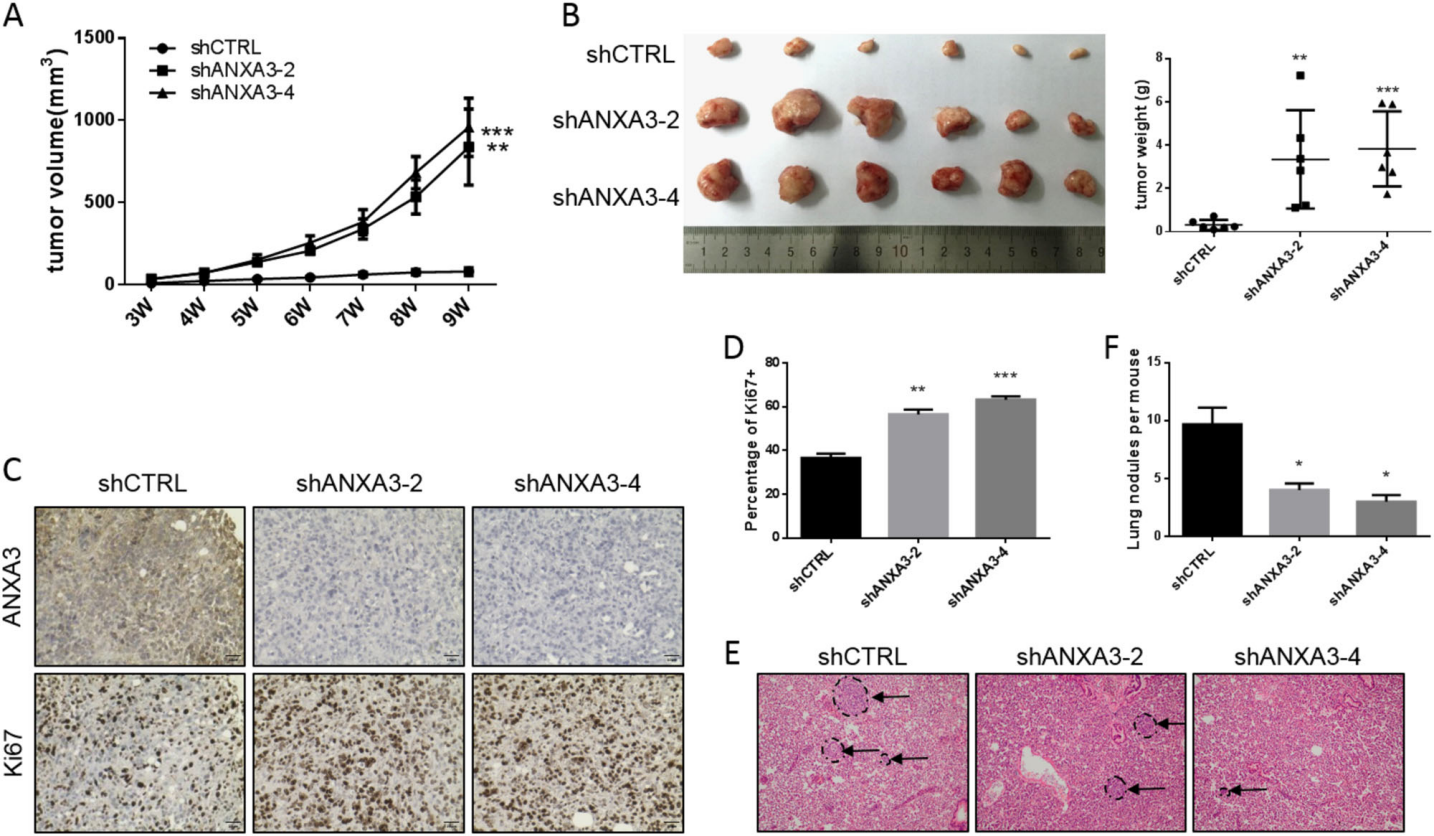
ANXA3 knockdown inhibits in vivo tumor metastasis but promotes tumor growth **a** For each group, 50,000 MDA-MB-231 cells were implanted into the mammary glands of 6- to 8-week-old female nude mice and tumor size was monitored weekly. **p < 0.01; ***p < 0.001. **b** The tumor images (left) and tumor weight (right) from **a** are shown. The tumor growth from **a** was halted when the largest tumor reached 15 mm in diameter for humane reasons. **p < 0.01; ***p < 0.001. **c** IHC staining of ANXA3 and Ki67 in the tumors from **b**. **d** Quantification of Ki67-positive cells (%) as shown in **c**. **p < 0.01; ***p < 0.001. **e** HE staining in slices of lungs harvested from mice in **a**. Arrow shows the metastatic nodules. **f** Quantitative analysis of the metastatic nodules in **e**. *p < 0.05

The in vitro studies above showed that *ANXA3* knockdown could significantly inhibit cell invasion but promote tumor cell proliferation. To further verify those results in vivo, 50,000 MDA-MB-231 cells with or without *ANXA3* knockdown were implanted into the mammary glands of 6- to 8-week-old female nude mice. The tumor size was monitored and measured weekly, and the results showed that *ANXA3* knockdown could significantly promote tumor growth (Fig. 3a). After the mice were sacrificed, tumors were harvested, photographed, and weighed. The tumors from the *ANXA3* knockdown group were significantly larger and heavier than those from the control group (Fig. 3b). Then, we stained the tumor sections for ANXA3 and Ki67 utilizing IHC staining and showed that ANXA3 was indeed downregulated in the *ANXA3*-knockdown group, and that *ANXA3* knockdown was correlated with much higher Ki67 expression, which characterizes cell proliferation activity (Fig. 3c, d). At the same time, lung slices of those mice were also subjected to hematoxylin-eosin (HE) staining, and the results showed that there were fewer metastatic nodules in the *ANXA3*-knockdown group (Fig. 3e, f). These in vivo results suggested that *ANXA3* knockdown promoted breast cancer tumor growth but inhibited metastasis.

### *ANXA3* inversely regulates two different states of breast cancer stem cells

Based on previous studies, BCSCs play a key role in tumor initiation and recurrence^27^. Our previous study showed that BCSCs exist in two alternative states: mesenchymal-like state BCSCs (CD24^−^/CD44^+^), which are relatively quiescent and have highly invasive capacity, and epithelial-like state BCSCs (ALDH^+^), which are associated with extensive proliferation^16^.

**Fig. 4:**
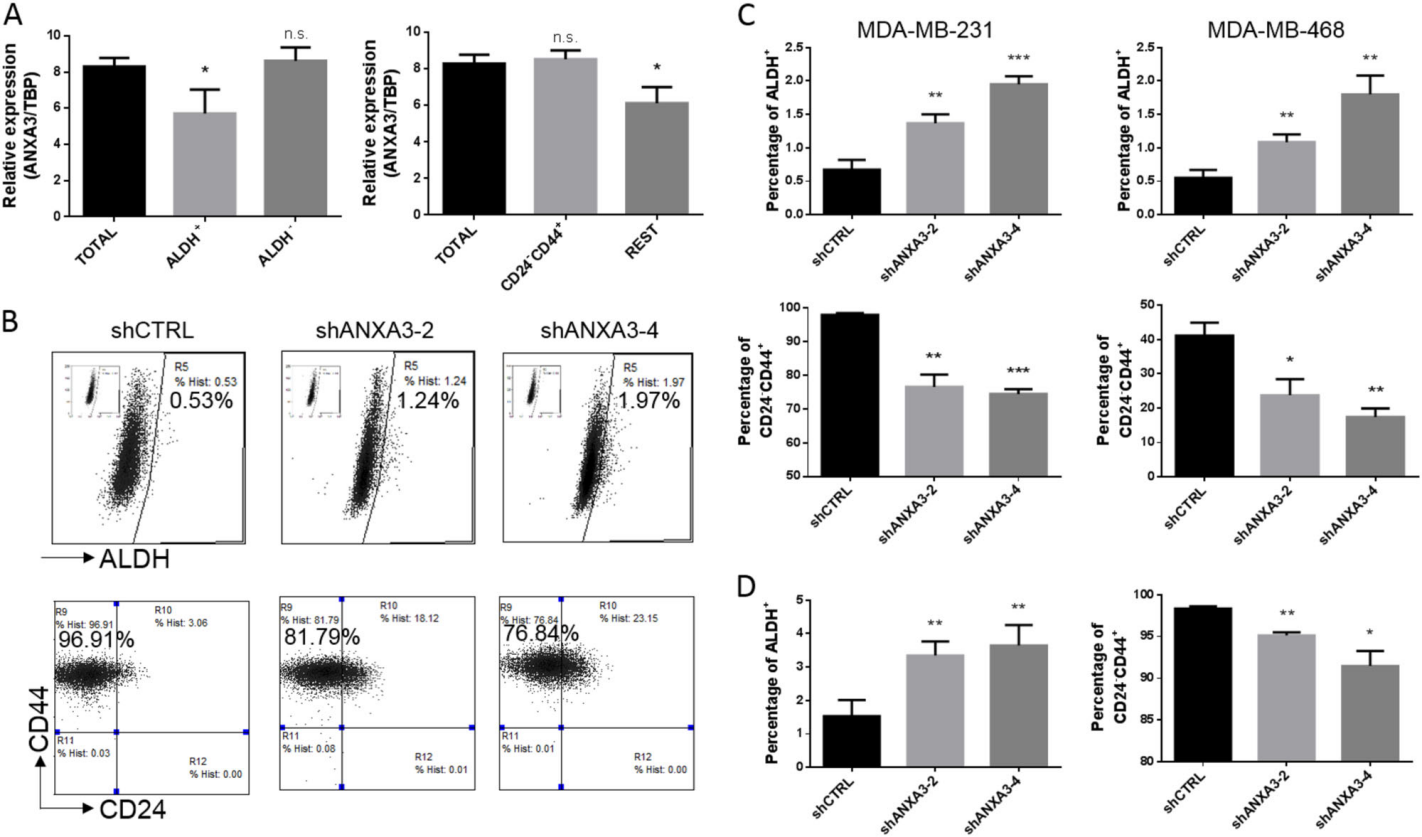
ANXA3 differentially regulates two states of breast cancer stem cells **a** The mRNA levels of ANXA3 were examined in FACS-sorted BCSCs from the MDA-MB-231 cell line by RT-PCR. *p < 0.05. **b** Flow cytometry analyses of BCSCs by the ALDEFLUOR assay (upper) and the CD24/CD44 assay (lower) in shCTRL- and shANXA3-infected MDA-MB-231 cells. **c** Quantification of ALDEFLUOR-positive or CD24^−^/CD44^+^ cells in shCTRL- and shANXA3-infected MDA-MB-231 and MDA-MB-468 cells. *p < 0.05; **p < 0.01; ***p < 0.001. **d** Quantification of ALDEFLUOR-positive or CD24^−^/CD44^+^ cells in digested tumor cells from Fig. 3b. *p < 0.05; **p < 0.01

To further characterize the role of *ANXA3* in BCSC regulation, we used two sets of markers for cell sorting by flow cytometry to obtain different subgroups of BCSCs in the MDA-MB-231 cell line. Then, we used qRT-PCR to detect the expression pattern of *ANXA3* in different subgroups of BCSCs (Fig. 4a). *ANXA3* showed a distinct expression pattern between the different subgroups. The expression of *ANXA3* was higher in the mesenchymal-like BCSCs (the CD24^−^/CD44^+^ population versus the non-CD24^−^/CD44^+^ population) but lower in the epithelial-like state (the ALDH^+^ population versus the ALDH^−^ population). Next, we examined the proportion of each BCSC population in the *ANXA3*-knockdown cell line, and we found that the CD24^−^/CD44^+^ population was decreased, but the ALDH^+^ population was increased (Fig. 4b, c, and S1). Similar findings were observed in the tumors from Fig. 3 (Fig. 4d). Taken together, these results suggest that *ANXA3* knockdown decreases the highly metastatic mesenchymal-like BCSCs but enriches the highly proliferative epithelial-like BCSCs, which might explain why the *ANXA3* knockdown inhibited cancer cell invasion and tumor metastasis but promoted cancer cell proliferation and tumor growth.

### The NF-κB signaling pathway plays a key role in *ANXA3*-regulated cancer cell proliferation and invasion

**Fig. 5:**
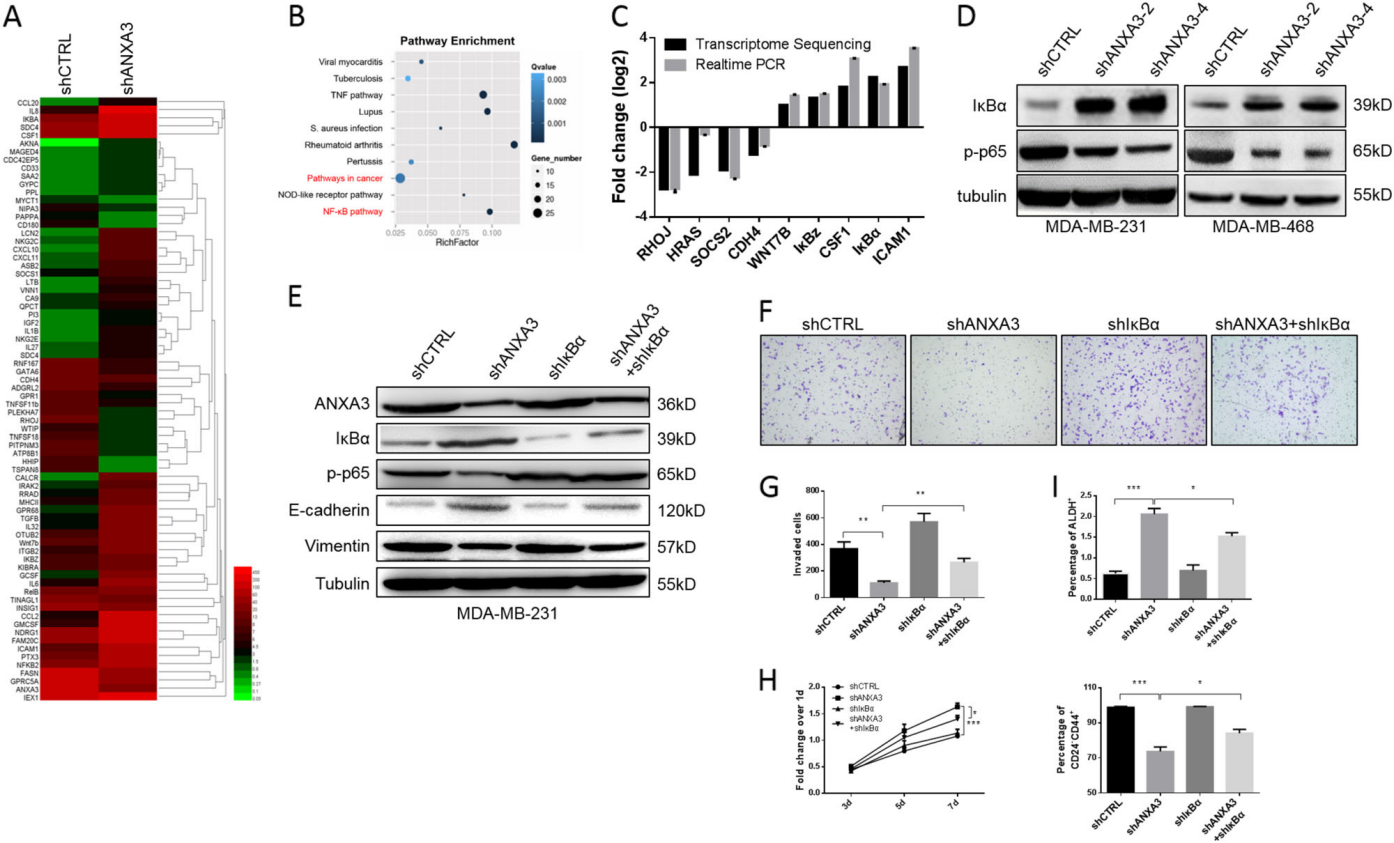
ANXA3-knockdown induces MET and affects breast cancer cell invasion and proliferation via inhibiting the NFκB pathway **a** Heatmap of gene expression in shCTRL- and shANXA3-infected MDA-MB-231 cells; each group has been repeated twice. **b** Pathway enrichment analysis of transcriptome profiling results from **a**. **c** Diagram showing the fold-changes of both the transcriptome sequencing and RT-PCR experiments of several typical genes to confirm and validate the sequencing data. **d** The protein levels of NFKBIA (IκBα) and phospho-p65 (p-p65) were detected by western blotting. **e** The protein levels of ANXA3, IκBα, phospho-p65, E-cadherin, and Vimentin were detected by western blotting. **f** Cell invasion ability was measured by the Matrigel invasion assay, as described in the methods. **g** Quantitative analysis of the total number of invaded cells in **f**. **p < 0.01. **h** Cell proliferation activity was measured by an MTT assay as described in the methods. *p < 0.05; ***p < 0.001. **i** Flow cytometry analyses of BCSCs by the ALDEFLUOR assay (upper) and the CD24/CD44 assay (lower) in shCTRL-, shANXA3-, shIκBα-, and shANXA3 + shIκBα-infected MDA-MB-231 cells. *p < 0.05; ***p < 0.001

To reveal the mechanisms of *ANXA3* knockdown-induced MET, *ANXA3*-knockdown and control cell lines were collected, and RNA-Seq was performed. Heatmap analysis showed that gene expression patterns are different between the control and *ANXA3*-knockdown groups (Fig. 5a). A scatter chart of the top ten enrichment pathways is shown (Fig. 5b), which suggests numbers of genes in the NF-κB signaling pathway were enriched in the profiling data. In addition, the mRNA expression of several significantly changed genes was confirmed by qRT-PCR (Fig. 5c). Considering IκBα (encoded by the *NFKBIA* gene) is a cellular protein that functions to inhibit the NF-κB transcription factor^28^, IκBα and phospho-NF-κB p65, an indicator of the activated canonical NF-κB pathway^29^, were analyzed by immunoblotting to validate NF-κB signaling pathway alteration (Fig. 5d). Increased IκBα and reduced p-p65 in the *ANXA3*-knockdown cells vs. scramble shRNA control suggested that the *ANXA3* knockdown inhibited the NF-κB pathway. To further investigate whether the NF-κB pathway plays a key role in the *ANXA3* knockdown-induced MET process, IκBα-knockdown cell lines with or without the *ANXA3* knockdown were established in the MDA-MB-231 cell line via a specific shRNA lentivirus infection. IκBα, p-p65, and mesenchymal/epithelial markers Vimentin/E-cadherin were detected by western blotting (Fig. 5e). The results showed that the MET state induced by the *ANXA3* knockdown was attenuated to some degree by the IκBα knockdown. The cell proliferation assay and cell invasion assay revealed that double knockdown of *ANXA3* and IκBα reduced cell proliferation and increased invasion compared to the *ANXA3* knockdown alone (Fig. 5f, h). Furthermore, the IκBα knockdown alone has little effect on either state of the BCSCs, but the IκBα knockdown partially abrogated the effects of the *ANXA3* knockdown on both the CD24^−^/CD44^+^ population and the ALDH^+^ population (Fig. 5i and S2). These results suggest that the NF-κB pathway played an important role in the *ANXA3* knockdown-induced MET process, in cancer cell proliferation and in cell invasion, and the IκBα knockdown abrogated the phenomenon caused by the *ANXA3* knockdown.

### The *ANXA3* knockdown enhances the efficacy of doxorubicin on tumor growth by increasing the uptake of doxorubicin in breast cancer cells

**Fig. 6:**
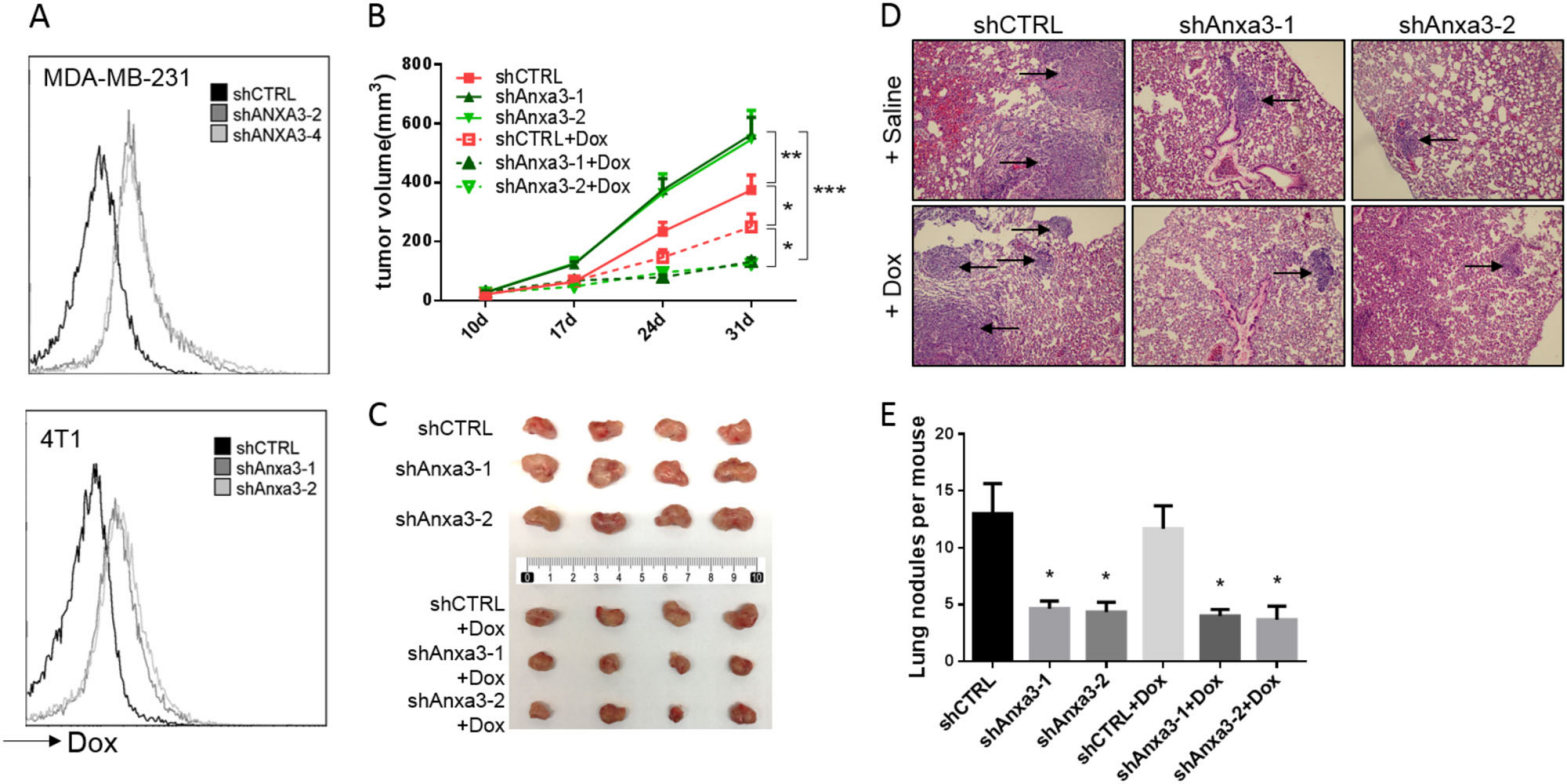
ANXA3 knockdown enhances the efficacy of doxorubicin on tumor growth and metastasis in vivo by increasing the uptake of doxorubicin in breast cancer cells **a** Flow cytometry analysis of MDA-MB-231 and 4T1 cells after incubation with doxorubicin for 4 h. The dose of doxorubicin was 5 μg/mL. **b** 4T1 cells (20,000) were implanted into the fourth mammary glands of 6- to 8- week-old female BALB/c mice, and the mice were divided into two groups for treatment: saline (control) and doxorubicin (Dox), and tumor size was monitored weekly. *p < 0.05; **p < 0.01; ***p < 0.001. **c** Mice were sacrificed at the end of treatment when the largest tumor reached to 15 mm in diameter for humane reasons, and the tumor image is shown. **d** HE staining in slices of lungs harvested from mice in **b**. Arrow show metastatic nodules. **e** Quantitative analysis of the metastatic nodules in **d**. *p < 0.05

As recent studies have shown that high *ANXA3* expression is involved in resistance to chemotherapy reagents in several cancers^21,22,24^, we investigated the effect of *ANXA3* on chemotherapy sensitivity in breast cancer cell lines. It has been shown that *ANXA3* reduced the accumulation of platinum in ovarian cancer cells^22^. Here, we analyzed the uptake of doxorubicin (Dox), which is widely used in breast cancer treatment, in *ANXA3*-knockdown cell lines by flow cytometry (Fig. 6a). Both human breast cancer cell line MDA-MB-231 and mouse mammary cancer cell line 4T1 showed an increased cellular accumulation of Dox in *ANXA3*-knockdown cells. Furthermore, we showed *ANXA3*-knockdown enhance the efficacy of both doxorubicin and docetaxel as the result of NF-κB pathway inhibition (Fig. S3A, B). Next, to ascertain whether the *ANXA3* knockdown influenced the sensitivity of breast cancer cells to Dox in vivo, the 4T1 tumor model was utilized. shCTRL cells and Anxa3 knockdown 4T1 cells were implanted to the fourth mammary glands of BALB/c mice, and each group was divided into two groups for different treatments when the tumor size reached approximately 2–3 mm in diameter: one group was treated with saline (control) and the other group was treated with Dox. After 4 weekly treatments, mice were sacrificed, and tumors were photographed and metastatic nodules in the lungs were counted. The results showed that *ANXA3* knockdown promoted breast tumor growth (Fig. 6b,c). However, tumor metastasis was reduced (Fig. 6d), as we have observed in the human breast cancer cell line xenografts. Interestingly, *ANXA3* knockdown enhanced the sensitivity of the cells to Dox, which antagonized the tumor-promoting effect from the *ANXA3* knockdown. And it has been reported that CD24^−^/CD44^+^ population are more resistant to chemotherapy, we determined the changes of CD24^−^/CD44^+^ BCSCs after Dox treatment with or without ANXA3 knockdown (Fig. S4). Those results indicated that DOX treatment induced an increase trend of CD24^−^/CD44^+^ BCSC population, which was eliminated by *ANXA3* depletion. These results suggested that the *ANXA3* knockdown enhanced the sensitivity of doxorubicin in breast cancer cells via increasing the cellular uptake, so the combination of *ANXA3* inhibition and doxorubicin therapy could inhibit tumor growth as well as tumor metastasis, which provides a novel combinational therapy approach to breast cancer.

## Discussion

ANXA3, a membrane associated protein, has been reported to be abnormally expressed in various cancers. *ANXA3* is highly expressed in lung, liver, and ovarian carcinoma and is correlated with a poor prognosis of patients^19–22^. However, *ANXA3* expression was essentially reduced in prostate and thyroid cancers, and the expression of *ANXA3* was negatively correlated with tumor development^30–32^. These studies suggest that *ANXA3* may play different roles in different tumors and reflect the heterogeneity and complexity of tumors^33^. However, *ANXA3* has not been well studied in breast cancer, and the biological role of ANXA3 is still unclear.

In this study, we found that *ANXA3* was significantly upregulated in breast cancer tissues at both the mRNA and protein levels, which were verified by qRT-PCR and immunohistochemical staining, respectively. *ANXA3* was also negatively correlated with patient prognosis in Kaplan–Meier survival analysis, which is consistent with a previous finding by Zeng et al.^34^.

In addition, we systematically studied the effect of *ANXA3* on breast cancer in vitro and in vivo, and this is the first investigation of the effect of *ANXA3* on BCSCs. Interestingly, others’ previous findings showed that *ANXA3* could simultaneously promote tumor cell proliferation and invasion in lung and liver carcinoma^25,35^, but we found that *ANXA3* knockdown inhibited tumor cell invasion but promoted cell proliferation both in vitro and in vivo. Our previous study showed the heterogeneity of BCSCs: the mesenchymal-like state (CD24^−^/CD44^+^) with a highly invasive capacity and the epithelial-like state (ALDH^+^) with an extensive ability to proliferate^16^. Currently, we showed that *ANXA3* knockdown induced MET and decreased the CD24^−^/CD44^+^ population but increased the ALDH^+^ population. Furthermore, previous studies showed that ANXA3 can promote CD133^+^ CSCs but there is no such meticulous distinction observed in the CSCs from lung or liver carcinoma, usually defined as the CD133^+^ population^25^, so the divergence between our current study and others’ studies in functional assays is understandable.

Next, we unraveled the mechanisms by which *ANXA3* knockdown induced the MET. Through gene expression profiling coupled with functional rescue experiments, we found that the NF-κB pathway was inhibited by ANXA3 knockdown. Multiple studies have shown that the NF-κB pathway is involved in the EMT^36^. Recent studies also found that NF-κB pathway activation regulates CD133^+^ CSCs^37–39^ and CD44^+^ CSCs^40,41^, but no studies have reported about the effect of the NF-κB pathway on ALDH^+^ CSCs. Our current study showed the NF-κB pathway regulation on the heterogeneity of BCSCs. Furthermore, IκBα-knockdown experiments showed effective but not complete rescue of the ANXA3-knockdown effect. Since, at the same time, we found that HRas and Wnt7b were also affected by the ANXA3 knockdown (Fig. 5a, b), the MAPK and Wnt pathways may also play a role in BCSC regulation by *ANXA3*, but more work is needed to validate this hypothesis.

As *ANXA3* is reported to assist in chemotherapy resistance^21,22,24^, our study focused on the therapeutic potential of targeting *ANXA3*. The increased cellular uptake of doxorubicin was observed in vitro when *ANXA3* was silenced. Considering that increased expression of annexin A1 and annexin A4 is also associated with drug resistance^42,43^, those members of the membrane-binding protein family may play important roles in chemotherapy resistance. Despite the fact that interfering with ANXA3 alone could significantly inhibit tumor metastasis with and cause increased tumor growth unexpectedly in vivo, targeting ANXA3 in combination with doxorubicin therapy could inhibit tumor growth and metastasis simultaneously. A study by Tong et al.^25^ showed that the original anti-*ANXA3* mouse antibody in combination with cisplatin exerted a synergistic inhibitory effect against hepatocellular carcinoma. Altogether, our results suggest that targeting *ANXA3* may be a promising novel treatment for tumor therapy in combination with the doxorubicin chemotherapy.

In conclusion, our study showed that there is a negative correlation between ANXA3 expression and breast cancer prognosis. ANXA3 could affect breast cancer cell proliferation and invasion by regulating the transition between mesenchymal-like state BCSCs and epithelial-like state BCSCs via the NFκB pathway. A combination of targeting ANXA3 and chemotherapy could provide a promising therapeutic approach to inhibit tumor growth and metastasis.

## Materials and methods

### Patients and clinical samples

The human breast cancer tissue used in this study, obtained from the First Affiliated Hospital of Anhui Medical University (Hefei, Anhui, China), was comprised of 16 pairs of breast cancer tissues and their corresponding adjacent normal tissues (Table S1). For each tissue sample, a piece was embedded with paraffin for an IHC assay, and another piece was ground with liquid nitrogen and then lysed with TRIzol (Thermo Fisher Scientific, New York, USA) to extract the total RNA for real-time PCR assay.

### Cell culture

Human breast cancer cell lines MDA-MB-231 and MDA-MB-468 and mouse breast cancer cell line 4T1 were purchased from ATCC. All of the cell lines were tested and authenticated shortly before use. These cell lines were cultured in RPMI-1640 medium with 10% fetal bovine serum (Thermo Fisher Scientific, New York, USA) and 1% streptomycin/penicillin (Beyotime, Shanghai, China) and maintained in a 37 °C atmosphere with 5% carbon dioxide (CO_2_).

### Short hairpin RNA plasmids and virus infection

shRNA plasmids were purchased from Sigma-Aldrich (St. Louis, Missouri, USA), and the effective sequences of ANXA3, Anxa3, and IκBα used in this study are described in Table S2. Knockdown (KD) lentiviruses were prepared by transfecting 293T cells; then, the cell lines were infected, and puromycin (Thermo Fisher Scientific, New York, USA) selection was performed for stable cell line establishment.

### RNA extraction and quantitative real-time PCR

Total RNA was extracted with RNAiso Plus (Takara, Beijing, China) and RNA concentration was measured with NanoDrop (Thermo Fisher Scientific, New York, USA). Complementary DNA (cDNA) was prepared from 1 µg RNA using the ReverTra Ace qPCR RT Kit (TOYOBO, Shanghai, China). qRT-PCR was carried out using AceQ qPCR SYBR Green Master Mix (Vazyme Biotech, Nanjing, China) in a real-time PCR system (7300, Applied Biosystems, New York, USA). TBP (TATA-box binding protein) was used as a reference gene. All primers used are shown in Table S3.

### Western blotting

Cells were collected and lysed in RIPA buffer containing a protease and phosphatase inhibitor cocktail (Roche, Mannheim, Germany) for 30 min on ice, and protein concentration was measured using a BCA Protein Assay Kit (Pierce, New York, USA). Then, samples mixed with 5x loading buffer were subjected to sodium dodecyl sulfate polyacrylamide gel electrophoresis. Proteins were transferred onto polyvinylidene fluoride (PVDF) membranes (Millipore, Billerica, USA) and incubated with the corresponding primary antibody and HRP-conjugated secondary antibody. The following antibodies and dilutions were used: anti-ANXA3 (1:200, sc-101885, Santa Cruz, Dallas, USA), anti-E-cadherin (1:1000, #20874-1-AP, Proteintech, Rosemont, USA), anti-N-cadherin (1:1000, #14215, CST, Danvers, USA), anti-γ-catenin (1:1000, #2309 s, CST), anti-Vimentin (1:1000, #5741s, CST), anti-IκBα (1:1000, #4812, CST), anti-phospho-p65 (1:1000, #3033, CST), anti-tubulin (1:1000, HC101-02, TransGen Biotech, Beijing, China), HRP-conjugated goat anti-mouse IgG (1:5000, sc-2005, Santa Cruz) and HRP-conjugated goat anti-rabbit IgG (1:5000, sc-2004, Santa Cruz). Chemiluminescent detection was performed using an ImageQuant LAS 4000 mini imaging system (GE, Fairfield, USA) with Western HRP Substrate (WBLUF0500, Millipore).

### MTT cell proliferation assay

Cells were seeded in 96-well culture plates at a density of 300–500 cells per well and cultured for 3, 5, or 7 days. MTT (Sigma-Aldrich) was added to a final concentration of 0.5 mg/mL, and the plates were incubated at 37 °C for 4 h. Then, 100 µL Dimethyl sulfoxide (DMSO) per well was added after removing the supernatants and shaking the plate for 10 min. The optical density at 490 nm (OD_490_) was measured with an Elx800 microplate reader (BioTek, Winooski, USA).

### Invasion assay

Twenty-thousand cells were seeded in Matrigel-coated (354234, Corning, New York, USA) Transwell chambers (8 µM Pore, 0216, BD, New York, USA), serum-free, with medium and 10% FBS in the bottom well. After culturing for 36 h, cells were fixed and stained with 0.1% crystal violet, and the invaded cells were photographed for statistical analysis.

### Flow cytometry

For the ALDEFLUOR assay (StemCell Technologies, Cambridge, USA), dissociated cells were suspended in assay buffer containing ALDEFLUOR substrate and incubated with or without aldehyde dehydrogenase inhibitor DEAB. A CD24/CD44 assay was performed with anti-CD24 (1:20, 561647, BD) and anti-CD44 (1:100, 560532, BD). For analysis of tumorigenesis in tumor cell suspensions, anti-mouse-lineage antibodies were used for H2Kd (1:100, 116607, Biolegend), CD45 (1:50, 555483, BD), CD31 (1:50, 555446, BD), CD140b (1:50, 558821, BD), and CD235a (1:50, 555570, BD). A MoFlo Astrios instrument (Beckman Coulter, Brea, USA) was used, and data acquisition and analysis were performed using Summit software.

### In vivo tumorigenesis

In this study, nude mice and BALB/c mice were utilized. All mice were bred and housed in AAALAC-accredited specific pathogen-free rodent facilities at the University of Science and Technology of China (Hefei, China). All mouse experiments were conducted according to standard operating procedures approved by the University Committee on the Use and Care of Animals at the University of Science and Technology of China. For the MDA-MB-231 tumorigenesis assay, 2 × 10^5^ cells were injected into the fourth mammary gland of 6- to 8-week-old female nude mice. In the 4T1 experiment, 2 × 10^5^ cells were injected into the fourth mammary gland of 6- to 8-week-old female BALB/c mice, and doxorubicin was administered at 5 mg/kg via intraperitoneal injection weekly. Tumors were monitored weekly until mice were sacrificed when the diameter of tumors reached 1.0–1.5 cm. Tumor volume was calculated as 1/2 × length × width^2^. For each tumor, one part was embedded in paraffin for histological analysis and the rest was digested into a single cell suspension using collagenase/hyaluronidase (Stem Cell Technologies) for flow cytometry. Lungs were embedded for HE staining and metastasis analysis.

### Immunohistochemistry

The slices of paraffin-embedded tissues were dewaxed and rehydrated in xylene and graded alcohol solutions. Anti-ANXA3 (sc-101885, Santa Cruz) and anti-Ki67 (1:200, ZA-0502, ZSGB-BIO, Beijing, China) were used to stain the slices. Cell nuclei were stained with hematoxylin (ZLI-9610, ZSGB-BIO).

### Gene expression profiling

Cells were collected, washed, and stored in RNAiso plus (Takara) at -80 °C, and gene expression profiling was performed at BGI (Shenzhen, China). Generally, after total RNA extraction and DNase I treatment, magnetic beads with Oligo(dT) were used to isolate mRNA, and the mRNA was fragmented into short fragments by mixing with the fragmentation buffer. cDNA was synthesized using the mRNA fragments as templates. After single nucleotide A (adenine) addition, the short fragments were connected with adapters. An Agilent 2100 Bioanalyzer and an ABI StepOnePlus Real-Time PCR System were used for quantification and qualification of the sample library. Finally, the library was sequenced using an Illumina HiSeq^TM^2000 platform or another sequencer when necessary.

### Statistical analysis

All values are presented as the mean ± s.e.m., except where otherwise indicated. Statistical analysis was performed using a two-tailed Student’s *t*-test with GraphPad Prism 6 (GraphPad Software, La Jolla, USA). A *p-*value < 0.05 was considered statistically significant.

The log-rank *p*-values of the overall survival analyses for breast cancer patients, the data of which was obtained from the Oncomine database (available at http://www.Oncomine.org), were computed using GraphPad Prism 6.

## Electronic supplementary material

SUPPLEMENTAL MATERIAL
