# Supplementary material for: Downregulation of annexin A3 inhibits tumor metastasis and decreases drug resistance in breast cancer
Source: Cell Death Dis. 2018 Jan 26;9(2):126. doi: 10.1038/s41419-017-0143-z (PMC5833718; doi:10.1038/s41419-017-0143-z)

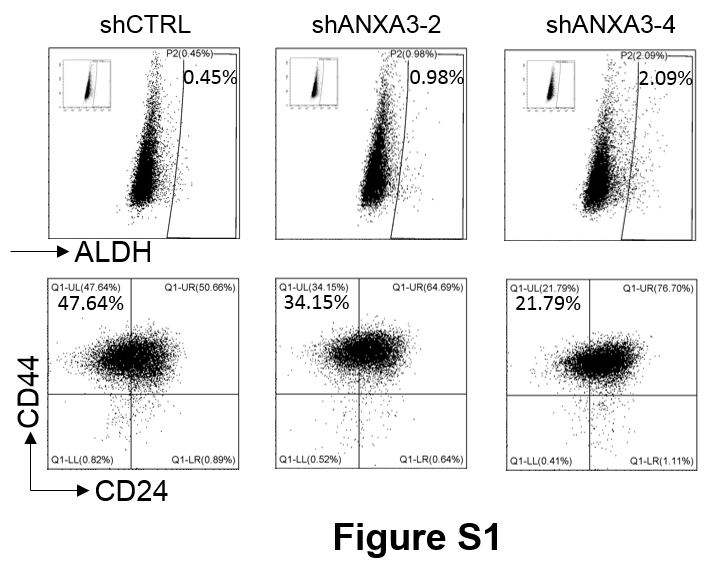


**Figure S1.** **ANXA3 regulates two states of breast cancer stem cells differently.** Flow cytometric analyses of BCSCs by ALDEFLUOR assay (upper) and CD24-CD44+ assay (down) in shCTRL and shANXA3 of MDA-MB-468 cells


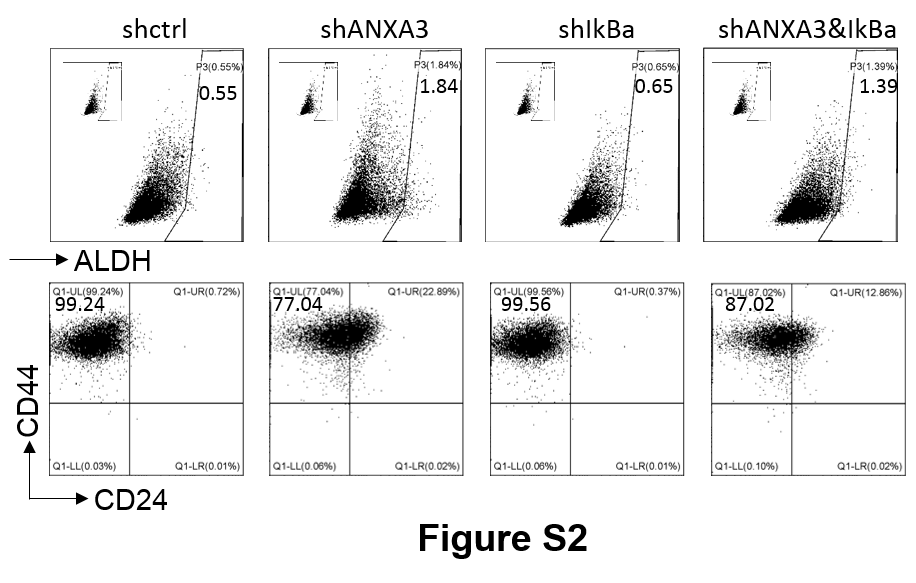


**Figure S2.** **ANXA3-knockdown affects breast cancer stem cells via inhibiting NFκB pathway.** Flow cytometric analysis of BCSCs by ALDEFLUOR assay (upper) and CD24-CD44+ assay (down) in shCTRL, shANXA3, shIκBα and shANXA3+shIκBα of MDA-MB-231 cells


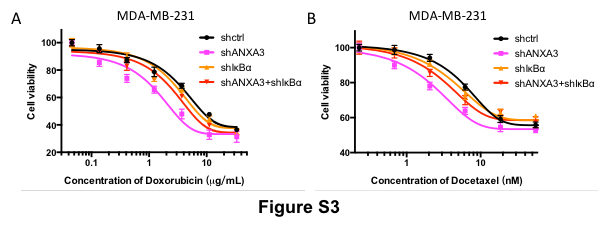


**Figure S3. ANXA3-knockdown enhances the efficacy of chemotherapy via inhibiting NF-κB pathway.** (A) MTT analysis of sensitivity to doxorubicin for shCTRL, shANXA3, shIκBα and shANXA3+shIκBα of MDA-MB-231 cells after being incubated with doxorubicin at indicated concentration for 3 days. (B) MTT analysis of sensitivity to docetaxel for shCTRL, shANXA3, shIκBα and shANXA3+shIκBα of MDA-MB-231 cells after being incubated with docetaxel at indicated concentration for 3 days.


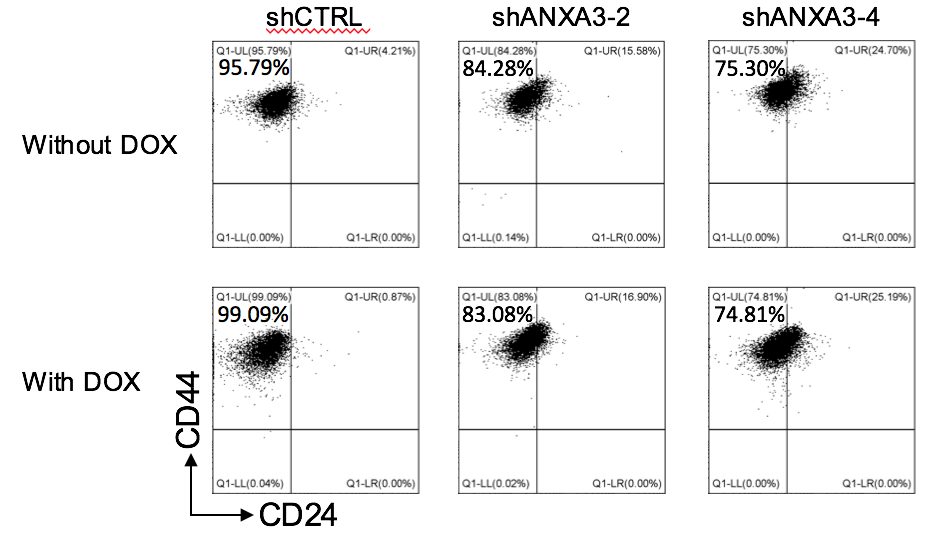


**Figure S4. ANXA3 knockdown eliminated the chemotherapy resistance of CD24^-^/CD44^+^ BCSCs.** Flow cytometry analyses of CD24^-^/CD44^+^ BCSCs in shCTRL and shANXA3 MDA-MB-231 cells with or without DOX treatment.


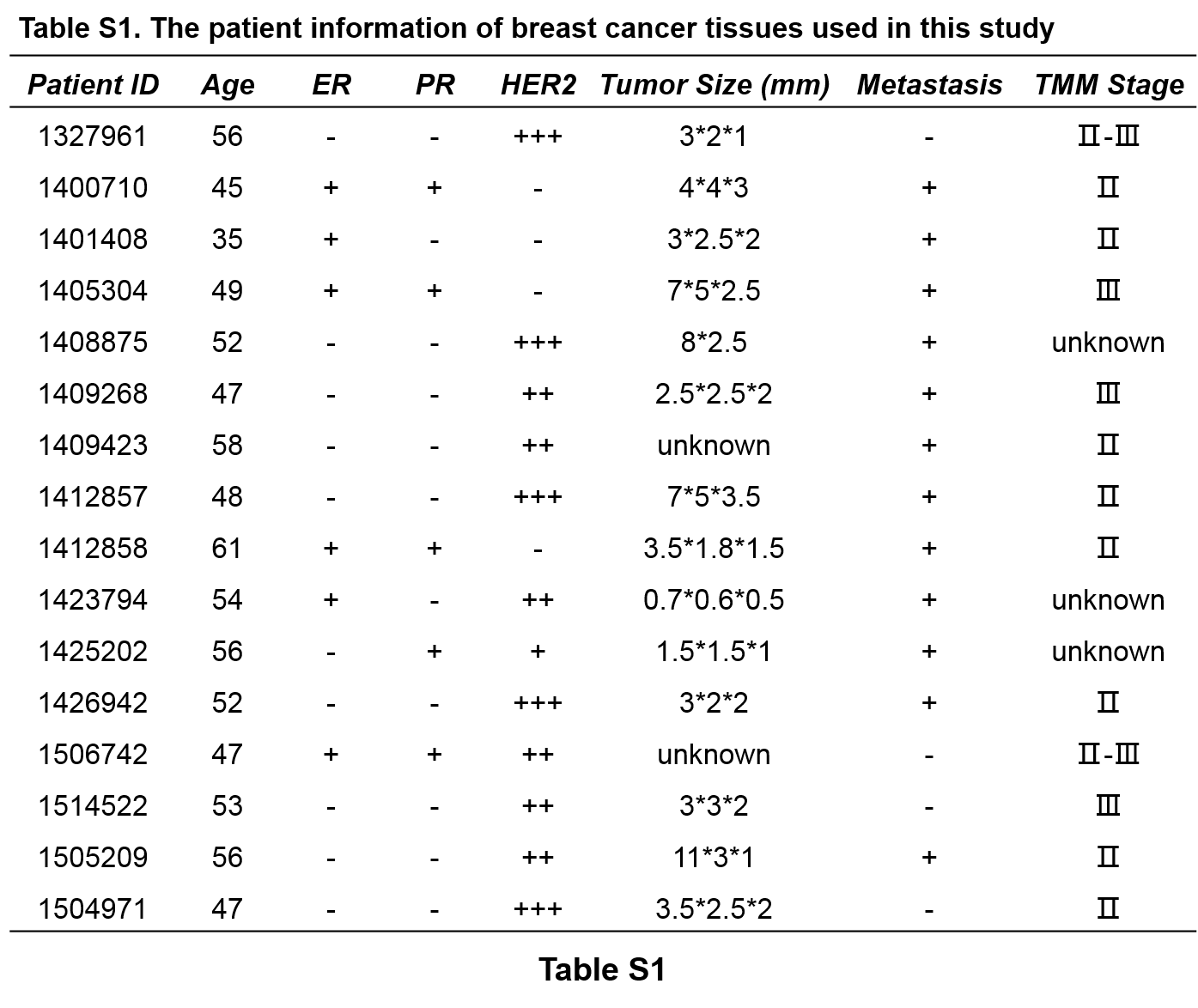


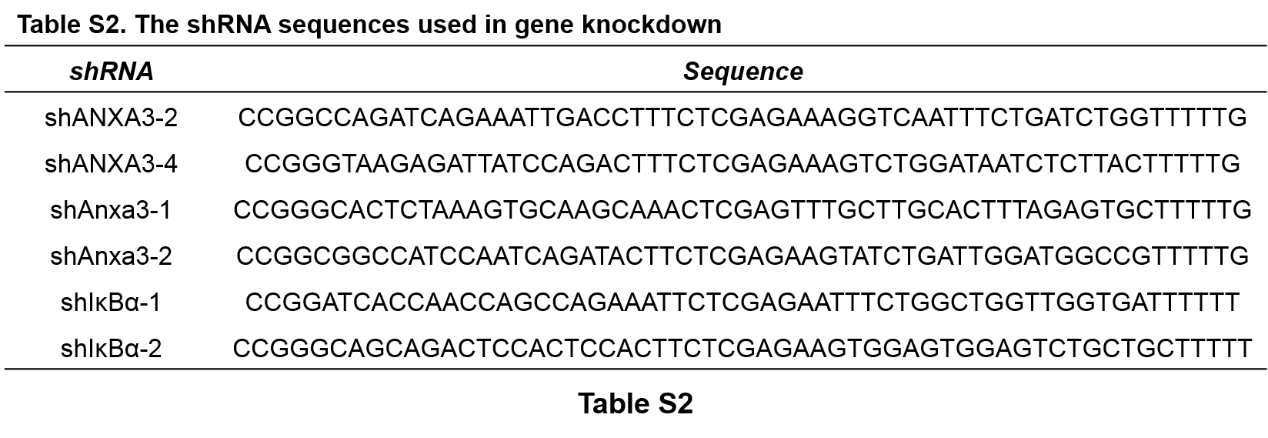


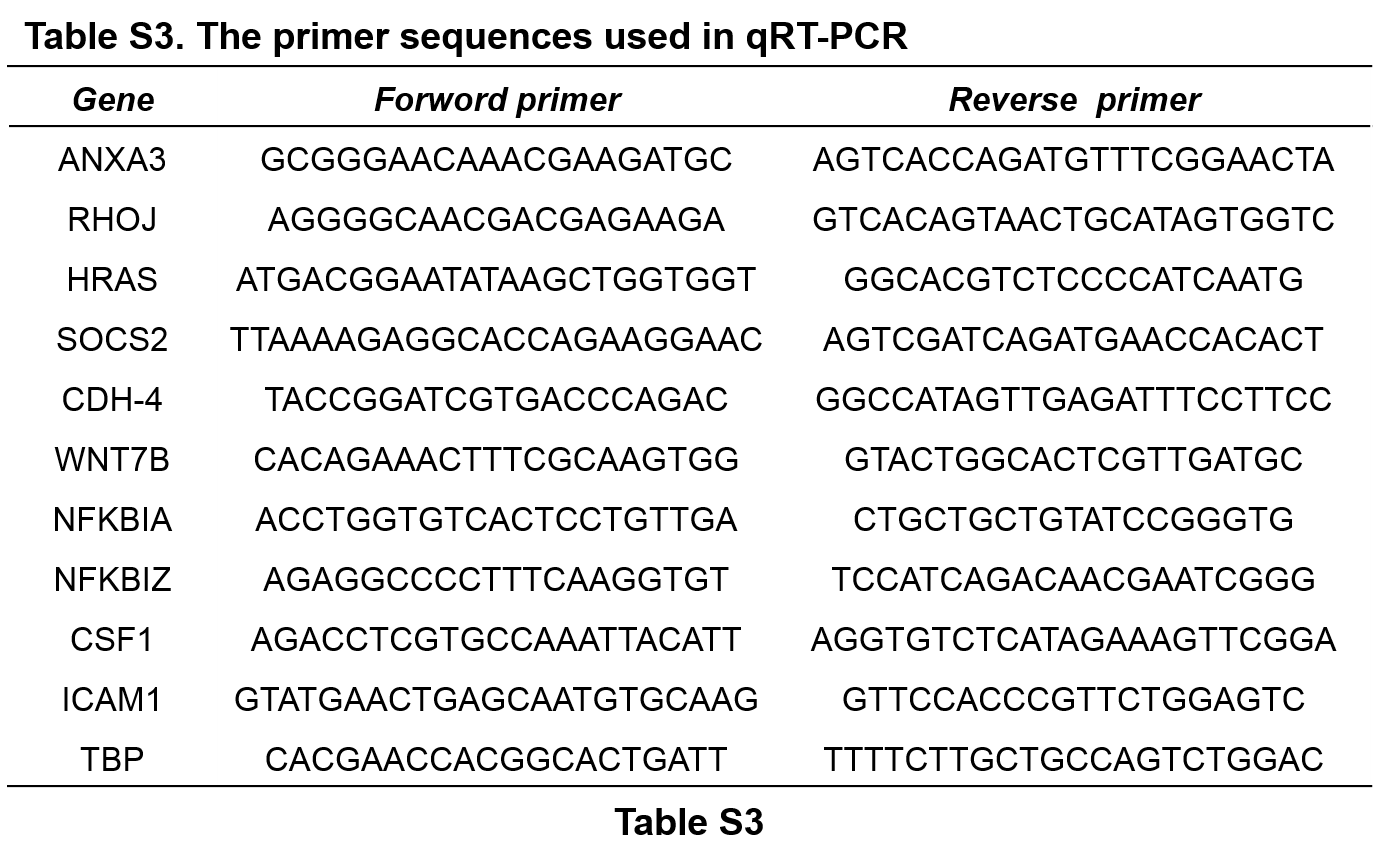

Supplement: Supplementary file 1 — SUPPLEMENTAL MATERIAL [file 41419_2017_143_MOESM1_ESM.docx]
